# Bismuth for Sweating Feet

**Published:** 1887-08

**Authors:** 


**Bismuth for Sweating Feet.**—M. Vieusse states that excessive sweating of the feet, under whatever form it appears, can be quickly and carefully conductəd friction with subnitrate of bismuth, and even in the few cases where this suppresses the abundant sweating only temporarily it still removes the fetidity which often accompanies the secretion.

